# From Unrealistic to Functional Optimism in Illness Perception: A Psychometric Comparison Across 10 Countries

**DOI:** 10.1111/sjop.13098

**Published:** 2025-04-30

**Authors:** Elisa Kern de Castro, Oscar Lecuona, Maria João Figueiras, Cristina Quiñones, Kamlesh Singh, Shoshana Shiloh, Michaela Schippers, Ana Kinkead, Raquel Rodríguez‐Carvajal

**Affiliations:** ^1^ Egas Moniz School of Health and Science Almada Portugal; ^2^ Universidad Autónoma of Madrid Madrid Spain; ^3^ Universidad Complutense de Madrid Madrid Spain; ^4^ Zayed University Abu Dhabi UAE; ^5^ Universidad de Burgos Burgos Spain; ^6^ Indian Institute of Technology Delhi Delhi India; ^7^ Tel Aviv University Tel Aviv Israel; ^8^ Erasmus University Rotterdam Rotterdam the Netherlands; ^9^ Universidad Autónoma de Chile Providencia Chile

**Keywords:** COVID‐19, illness perception, optimism, perceived risk

## Abstract

People's perceptions of illness and its risks influence health behaviors, including risk management and precautionary measures. Illness perception often involves unrealistic optimism, reducing infection risk perception. However, crises disrupt self‐regulation and optimism due to uncontrollable situations. This study examines optimism's link to risk and illness perception during the first COVID‐19 wave in 10 countries, with 7254 participants (48.1% women, mean age = 40, SD = 14.8). We used Bayesian structural equation modeling for psychometric stability and one‐way ANOVAs for country comparisons. Multiple regression analyses examined the impact of optimism and demographic variables on illness perception. Significant cross‐country variations emerged in illness perception and optimism. In terms of the relationship between variables, optimism correlated with increased COVID‐19 risk perception, especially for negative outcomes, concern, and consistency. During crises, optimism shifted from unrealistic to functional, promoting treatment adherence, personal control, and coherence. These dimensions represent individuals' beliefs in managing illness, highlighting optimism's adaptive role in crises.

**Summary**

- Study explores optimism's role, traditionally seen as a risk factor for underestimating health threats.
- Optimism positively correlates with COVID‐19 risk perception, challenging expectations.
- Results reveal optimism's functional role in enhancing perceived control and illness coherence.
- Pessimism is associated with greater emotional impact from COVID‐19 and longer illness timelines.
- Key findings on this new optimism's role are consistent across ten countries.

## 1 Introduction

Studying how individuals and groups behave in the presence of illnesses is a relevant topic of psychological research. This is due to the potential to explain reactions to these events, which can be studied as “health behaviors.” To study them, prior research points out two relevant concepts: (1) illness perceptions and (2) risk perceptions.

Illness perceptions are mental representations of illnesses. According to the Common‐Sense Model of health and illness (CSM), illness perceptions involve evaluating different aspects of illnesses, considering individual and social experiences (Leventhal et al. 1984, 2012). Core components of the model are the subjective perceptions about illness, such as its symptoms, causes, timeline, patient and treatment control, consequences, and emotional impact of the illness. Thus, when individuals perceive a threat to their health status, they construct their mental models. These mental models have flexible cognitive schemas that integrate information from cultural and social contexts and previous direct and indirect experiences with illnesses. Mental models guide people in making decisions about and coping with the threat (Leventhal 2019; Leventhal et al. 2016). Illness perceptions hold the potential to explain relevant health behaviors such as risk management, adherence to prescriptions, and quality of life (Broadbent et al. 2015; Hagger and Orbell 2003; Leventhal et al. 2016).

In contrast, risk perceptions are a subjective psychological construct influenced by cognitive, emotional, social, cultural, and individual variations among individuals and countries (Dryhurst et al. 2020). Risk perceptions are a crucial predictor of health behavior, according to psychological theories such as the Health Belief Model (Maiman et al. 1977) or the Theory of Reasoned Action (Fishbein et al. 2007). They provide insight into how beliefs and attitudes shape behavior. Although risk perceptions are perceptions of absolute personal risk, the construct is inherently comparative (Klein 2003). When faced with threats, individuals evaluate their level of danger by comparing their own risk to the average risk of their peers. In some contexts, there is some evidence that people tend to underestimate their probability of infection when comparing themselves to others like them (Bottemanne et al. 2020; Monzani et al. 2021; Shiloh et al. 2021). On a group level, this is called “unrealistic optimism” (Radcliffe and Klein 2002). Recent research indicates that dispositional optimism may increase the tendency to engage in comparative unrealistic optimism (Bottemanne et al. 2020). From a psychological perspective, optimism is considered a personality trait, in which people tend to have general positive expectations about their future (Carver and Scheier 2014). In situations beyond our control, more optimistic individuals might be more prone to underestimate the risk of threat, both in absolute terms and comparatively. The COVID‐19 pandemic serves as a recent example of such scenarios.

The COVID‐19 pandemic is considered one of the most significant global crises since World War II (United Nations 2020). Next to the human death toll of the crisis, the measures taken also seem to have had a major economic and social impact (Herby et al. 2022; Schippers et al. 2022). In this context, behavioral science has shown to be effective regarding adherence to policies of preventive measures (Bonfanti et al. 2023). Positive social norms and trusted leaders have empirical support for promoting a functional perception and adherence to preventive measures. Human behavior plays a crucial role in preventing the spread of any virus. It is essential to understand people's subjective perceptions of illness, risk, and resulting prevention behaviors. Mental models of illness and risks associated with COVID‐19 may influence their preventive behaviors. Prior research has shown the relevance of illness and risk perceptions toward COVID‐19 toward predicting threat‐relevant behaviors (Chong et al. 2020; Man et al. 2020; Peleg et al. 2016; Skapinakis et al. 2020). Other studies have examined how illness and risk perceptions are relevant in predicting psychosocial variables, such as non‐pharmaceutical interventions (Dennis et al. 2021; Gabanelli et al. 2024; Vindegaard and Benros 2020). Perhaps the most significant impact of risk perception is the positive associations with adherence to preventive health behaviors, such as handwashing, as found in a study conducted across 10 countries (Dryhurst et al. 2020). Studies have found that concrete aspects of illness perceptions, such as direct and indirect experience with the disease, belief in science (Bonfanti et al. 2023; Wingen et al. 2022), and low collective efficacy or prosocial values are associated with adherence to preventive behaviors. Other studies have found a positive association between illness and risk perceptions and adherence to preventive behaviors. This phenomenon has been observed in various populations during the early stages of COVID‐19, including Chinese (Chong et al. 2020), European (e.g., Skapinakis et al. 2020), Mexican (Lugo‐González et al. 2020), and Israeli populations (Shiloh et al. 2021). Overall, these studies suggest that COVID‐19 is often perceived as a highly contagious illness with severe consequences and limited personal control.

While unrealistic comparative optimism can occur at various levels of absolute perceived risks, some studies have found that high levels of optimistic bias about COVID‐19 were associated with a lower perceived risk of infection, which, in turn, would hinder preventive behaviors (Monzani et al. 2021; Park et al. 2021; Radcliffe and Klein 2002). Although COVID‐19 was generally perceived as a highly contagious illness, highly optimistic individuals might display a lower adherence to preventative measures, damaging their personal and collective health. Conversely, pessimism would display the opposite pattern, such as higher adherence to preventive measures. In situations of high risk and illness perception, individuals with high levels of pessimism may be more prone to adopt increasingly intense preventive measures due to their expectation of worse outcomes.

The first wave of COVID‐19 provided a unique opportunity to compare illness perceptions and risk perceptions of this disease across various countries and to contrast how illness perceptions, risk perceptions, and dispositional optimism and pessimism are associated, since at that time a vaccine was not developed and risk perceptions were high. Research on these associations would provide potential explanatory models for future outbreaks or pandemics. More concretely, the role of dispositional optimism would be of particular importance in understanding illness and risk perceptions.

The aims of this study were as follows: (a) to compare illness perceptions, absolute and comparative risk perceptions of COVID‐19, and dispositional optimism and pessimism among ten countries; (b) to investigate the associations between dispositional optimism and risk perceptions toward illness perceptions of COVID‐19 in 10 countries. Our hypotheses are as follows: (a1) dispositional optimism and pessimism would provide stable psychometric properties across countries, (a2) illness perceptions would be approximately high in consequences and concern, and low in levels of control across countries, (a3) risk perceptions would be high across countries, and (b) dispositional optimism and risk perceptions toward COVID‐19 would be negatively associated in all 10 countries.

## 2 Materials and Methods

### 2.1 Sample

An overall sample of 7254 participants was obtained by merging several samples from Peru (*N* = 3343), France (*N* = 950), the United Kingdom (*N* = 628), India (*N* = 611), Portugal (*N* = 549), Spain (*N* = 479), Israel (*N* = 258), the Netherlands (*N* = 172), Chile (*N* = 134), and Germany (*N* = 130). All samples but one (India) counted most participants as middle‐aged (around 40 years). All samples but two (Portugal and Peru) were predominantly female (around 60%). Chronic disease was minor (around 5%) except for two samples (France and Germany), which had higher values. Concerning COVID‐19 measures, most countries had active lockdowns except for Portugal, the Netherlands, the United Kingdom, France, and Germany. However, social isolation adherence was frequent (around 60%) except in countries with absent or low lockdowns (Portugal, Netherlands, United Kingdom, Israel, France, and Germany). The only exception to this pattern was India, with the highest lockdown (almost 13 days at the time of data collection) but with one of the lowest social isolation adherences (2.4%). Finally, COVID‐19 incidence measures were heterogeneous. While admitted COVID‐19 infection was low (around 25% except in Spain and India, with lower values), family members were reported to have frequent contagions. This is because around 70% of participants reported having at least one family member infected with COVID‐19. The only exceptions were Portugal and Spain, with lower incidences (around 35%). Finally, COVID‐19 incidence among friends was the lowest of all (around 10%), with the only exception of Spain, with a higher value (around 50%). Table 1 displays detailed demographic variables.

**TABLE 1:** Descriptive statistics for demographic variables for each country and overall samples.

Sample	Age	% Female	Chronic disease (% yes)	Days of lockdown	Social isolation (% yes)	COVID‐19 infection (% yes)		
						Self	Family member	Friend
Overall (*N* = 7254)	40 (14.8)	48.1	13.4	1 (1.5)^a^	64.1	24.3	69.1	10.6
Portugal (*N* = 549)	42.1 (11.9)	20.1	5.7	—	7.4	25.9	31.1	1.5
Peru (*N* = 3343)	42.1 (11.9)	33.8	0.5	1 (1.5)^a^	85.3	16.3	65.4	1.5
Chile (*N* = 134)	39.9 (10.8)	69.7	9.4	2.1 (1.5)^a^	78.8	32.6	66.7	3.1
Netherlands (*N* = 172)	38.9 (10.8)	74.7	3.3	2 (0.1)^a^	—	22.5	84.1	10.6
Spain (*N* = 479)	42.4 (13.5)	72.6	19.3	2 (1)^a^	73.6	5.5	38.2	50.5
United Kingdom (*N* = 628)	45.2 (16.2)	51.2	0.8	0.1 (0.1)	6.2	25.5	82.8	5.4
India (*N* = 611)	22 (4.4)^a^	63.3	0.2	12.8 (8.5)	2.2	7.1	97.3	0.8
Israel (*N* = 258)	55.9 (15.6)	64.9	1	3 (1.5)^a^	2.4	32.9	87.7	5.16
France (*N* = 950)	45 (13.3)^a^	87.2	40.7	—	—	19.2	93.1	16.1
Germany (*N* = 130)	38.9 (14.7)	79.2	72.4	—	—	13.2	91.4	5.7

*Note:* Bracketed with each descriptive are standard deviations (continuous variables).

^a^ Median and median absolute deviation due to skewness>|1|.

### 2.2 Instruments

#### 2.2.1 Sociodemographic Questionnaire

We designed a short questionnaire to assess relevant sociodemographic characteristics alongside potentially relevant COVID‐19 variables. Sociodemographic variables were age and gender. Health‐relevant variables were the presence of chronic disease, several days in lockdown, practicing social isolation (and the number of days), having been infected with COVID‐19, and having infected family members or friends.

#### 2.2.2 Brief Illness Perception Questionnaire (B‐IPQ)

The B‐IPQ measures illness perceptions, which are mental representations that people have about an illness (Broadbent et al. 2006). It consists of eight items with a Likert response format (0 = not at all; 10 = totally), each measuring a separate sub‐scale of the Common‐Sense Model of illness perceptions (Leventhal et al. 2012). The items measure: consequences (illness' effect on a patient's life), identity (how much a patient experiences symptoms of the illness), timeline (how long does the illness last), personal control (patient's control over the illness), treatment control (treatment can help the patient's illness), illness comprehensibility (how well the illness understood), concern about the illness, and its emotional representations. An additional item has an open‐ended response format, measuring up to three perceived causes of the assessed illness. A full display of the B‐IPQ is available on the OSF platform. An extensive review of 188 scientific studies using the B‐IPQ showed good psychometric properties with a wide range of illnesses, in 26 languages from 36 countries. Illness perceptions measured by the B‐IPQ were associated with important physical health outcomes including survival/mortality rates (Broadbent et al. 2015).

#### 2.2.3 Life Orientation Test (LOT‐R)

The LOT‐R measures dispositional optimism and pessimism (Scheier et al. 1994). It consists of items such as “I am always optimistic about the future,” with a Likert response format (1 = totally disagree; 5 = totally agree). A total score is obtained by averaging across the items. As we recruited several countries for our study, we used their respective validated versions of the LOT‐R. This led to the implementation of two versions of the LOT‐R: Most countries used the 15‐item LOT‐R (e.g., Herzberg et al. 2006), while the samples of Portugal, France, and Germany used the 10‐item LOT‐R (e.g., Laranjeira 2008).

#### 2.2.4 Absolute and Comparative Risk Perceptions

These measures were adapted from previous studies on risk perception in non‐clinical samples (Dryhurst et al. 2020; Figueiras et al. 2017; Gerhold 2020). Two items with a Likert response format (0 = not at all; 10 = extremely high) were used. Absolute risk perceptions: “To what extent do you feel at risk of contracting this illness over the next 12 months?”, and comparative risk perceptions: “Compared to people of your same age and gender, to what extent do you feel at risk of contracting this illness over the next 12 months?”

### 2.3 Procedure

The data included samples from the general population of residents of the UK, Perú, Chile, Portugal, Spain, India, Israel, Netherlands, France, and Germany. Each country implemented the survey in their official languages while using the validated versions of the psychometric instruments (i.e., the B‐IPQ and the LOT‐R). A web‐based snowball sampling was used in all countries using Qualtrics and Google Forms on several social media. This included asking participants to post the study link on their social media to recruit participants. We excluded participants younger than 18 years old or those who were not residents of the country sampled. For the United Kingdom sample, quotas were implemented for gender (around 50% of male and female) and age (12% for 18–24 years old, around 20% in the ranges of 25–34, 35–44, 45–54 and 55–64, and 14% for > 65 years old). The research protocol was authorized by the Human Research Ethics Committee (HREC) of the Open University (HREC/3543). All participants provided informed consent by responding to the emailed survey. Confidentiality and the right to withdraw from the study were assured.

### 2.4 Analysis

Descriptive statistics were computed for the overall sample and each country. Given the presence of multiple groups, traditional techniques (e.g., Confirmatory Factor Analysis) were discarded in favor of a Bayesian structural equation modeling (BSEM, Muthén and Asparouhov 2012) to allow minor deviations from models as negligible. More concretely, we implemented an approximate invariance approach via multigroup BSEM (Lek et al. 2018, applied for example in Graeff Buhl‐Nielsen et al. 2020). In short, multigroup BSEM allows for small deviations between groups that are considered negligible, which is commonplace when dealing with multi‐country samples. This makes comparison more precise since traditional techniques such as CFA can become too strict when detecting differences. Minimally informative prior distributions for parameters were included to implement these models: the priors for factor loadings were specified as N(0.50,10). Item intercept priors were specified following the Likert structure as N(3,1) truncated from 1 to 5 (due the metric of variables being from 1 to 5 as they were item means). Factor intercept priors were specified as N(0,1). Regarding cross‐loadings, we selected incrementally informative priors, starting from low informative priors (e.g., N(0,1)) to highly informative priors (e.g., N(0,0.001)). These selected priors were previously applied in other empirical studies (Merkle and Wang 2018). When cross‐loadings were not applicable (e.g., unidimensional models), incrementally informative priors were specified for factor loadings as N(0.4, SD), with SD being incrementally lower. To study approximate invariance, two sets of models were produced. First, factor loadings across groups were specified with incrementally informative priors (i.e., from low informative priors such as N(0,1) to highly informative priors such as N(0,0.001)). This set of models was labeled as “metric” approximate invariance. Secondly, item intercepts across groups were specified with the same incrementally informative priors. This set of models was labeled as “scalar” approximate invariance. For all models, fit measures were the discrepancy information criterion (DIC), the Bayesian information criterion (BIC), and the posterior predictive *p* value (PPP). For the DIC and BIC, smaller values will indicate a better fit, while values < 0.05 will indicate a good fit for the PPP. However, the PPP seems to fail with complex models (Asparouhov and Muthén 2017; Cain and Zhang 2019), so zero or close‐to‐zero values were expected.

Once establishing psychometric stability, B‐IPQ item differences by country were assessed via a one‐way ANOVA. If assumptions were not met, corrections were implemented. For simplicity, we only reported marginal means with 95% confidence intervals as plots (detailed tests available in Supporting Informations). Power analysis with 95% confidence and 95% power revealed a minimum sample size of approximately 100 per group in pairwise comparisons for medium effect sizes (*d* = 0. 5), which was achieved for the worst case (Chile and Germany, *n* = 134 and *n* = 130, respectively). Finally, to estimate the relative importance of optimism, pessimism, and demographic variables regarding the B‐IPQ, we implemented multiple regression analyses (MRA). Power analysis with 95% confidence and 95% power and 11 predictors revealed a sample of 5652 allowing observable *R* ^2^ up to 0.004 and standardized coefficients (*β*) of 0.002. Our sample of 1629 allowed observable *R* ^2^ up to 0.015 and *β* of 0.006. All our *R* ^2^ and estimated *β* were larger than these criteria, thus making our sample size adequate. We estimated MRAs only for the 15‐item version of the LOT‐R (*n* = 5625) and not for the other with the 10‐item LOT‐R (*n* = 1629) due to psychometric results (see Section 3). Each B‐IPQ item was set as the dependent variable, while optimism, pessimism (only for the 15‐item LOT‐R), and demographic variables were set as predictors.

Data are available at the Open Science Framework (OSF): https://osf.io/pdwj2/. Descriptive statistics, ANOVAs, and MRAs were computed with Jamovi (The Jamovi Project 2023), while BSEM and approximate invariance analyses were computed with MPlus 7 (Muthén and Muthén 2012).

## 3 Results

### 3.1 Bayesian SEM

The BSEM and approximate invariances analyses found different conclusions for each version of the LOT‐R (Table 2). Regarding the 15‐item version, BSEM showed cross‐loadings very proximate to zero (best model fit for priors with SD = 0.01), thus displaying a good fit. Approximate invariance displayed low differences across samples in loadings (best model fit for priors with SD = 0.1), while medium differences in item intercepts (best model fit for priors with SD = 0.5). Hence, we interpreted the 15‐item LOT‐R as approximately metrically invariant, allowing for comparisons across groups, while potential differences in item means could appear in subsequent analyses. Regarding the 10‐item version, BSEM showed loadings very close to 0.4 (best model fit for priors between SD = 0.05 and 0.01), thus indicating a good fit. However, approximate invariance displayed considerable differences across samples in loadings (best model fit for priors with SD = 1). In addition, considerable to medium differences arose in item intercepts (best model fit for priors with SD between 1 and 0.5). Hence, the 10‐item LOT‐R presents considerable differences across samples, impeding comparison of scores between them. We proceeded to consider the LOT‐R measures only for samples with the 15‐item version in subsequent analysis (i.e., ANOVAs and MRAs).

**TABLE 2:** Fit measures of all models of LOT‐R in BSEM and approximate invariance analyses.

Model	Prior SD	DIC	BIC
15‐item LOT‐R			
BSEM	1	214,458.40	214,873.46
	0.5	**214,405.61**	215,179.48
	0.1	214,459.57	214,870.4
	0.05	214,460.5	214,870.61
	**0.01**	214,460.55	**214,870.51**
	0.001	214,514.03	214,929.78
Approximate metric invariance	1	**203,871.66**	206,892.87
	0.5	204,051.11	206,888.83
	**0.1**	204,359.97	**206,881.67**
	0.05	204,437.51	206,885.54
	0.01	204,664.16	207,088.581
Approximate scalar invariance	1	**204,359.97**	206,881.69
	**0.5**	204,361.87	**206,879.02**
	0.1	204,372.56	206,907.8
	0.05	204,382.51	206,925.19
	0.01	204,494.81	207,063.43
10‐item LOT‐R			
BSEM	0.5	**22,968.95**	23,443.54
	0.1	23,240.24	23,443.57
	**0.05**	23,324.09	**23,443.55**
	**0.01**	23,354.68	**23,443.71**
	0.001	23,370.82	23,459.87
Approximate metric invariance	1.5	**20,347.91**	20,669.77
	1	20,353.94	**20,669.74**
	0.5	20,364.12	20,670.37
	0.1	20,383.14	20,672.33
	0.05	20,388.48	20,673.09
	0.01	20,406.6	20,689.57
	0.001	20,291.86	20,758.27
Approximate scalar invariance	1.5	**20,338.73**	20,671.98
	**1**	20,353.94	**20,669.74**
	**0.5**	20,367.24	**20,670.11**
	0.1	20,371.7	20,675.22
	0.05	20,379.85	20,690.01
	0.01	20,499.89	20,826.8
	0.001	20,615.02	20,957.84

*Note:* Bolded are the lowest DIC and BIC values, suggesting the best fit. All models displayed *p* < 0.001 and achieved simple structure.

### 3.2 Comparisons of Illness Perceptions, Absolute and Comparative Risk Perceptions, and Dispositional Optimism Among Different Countries

**FIGURE 1:**
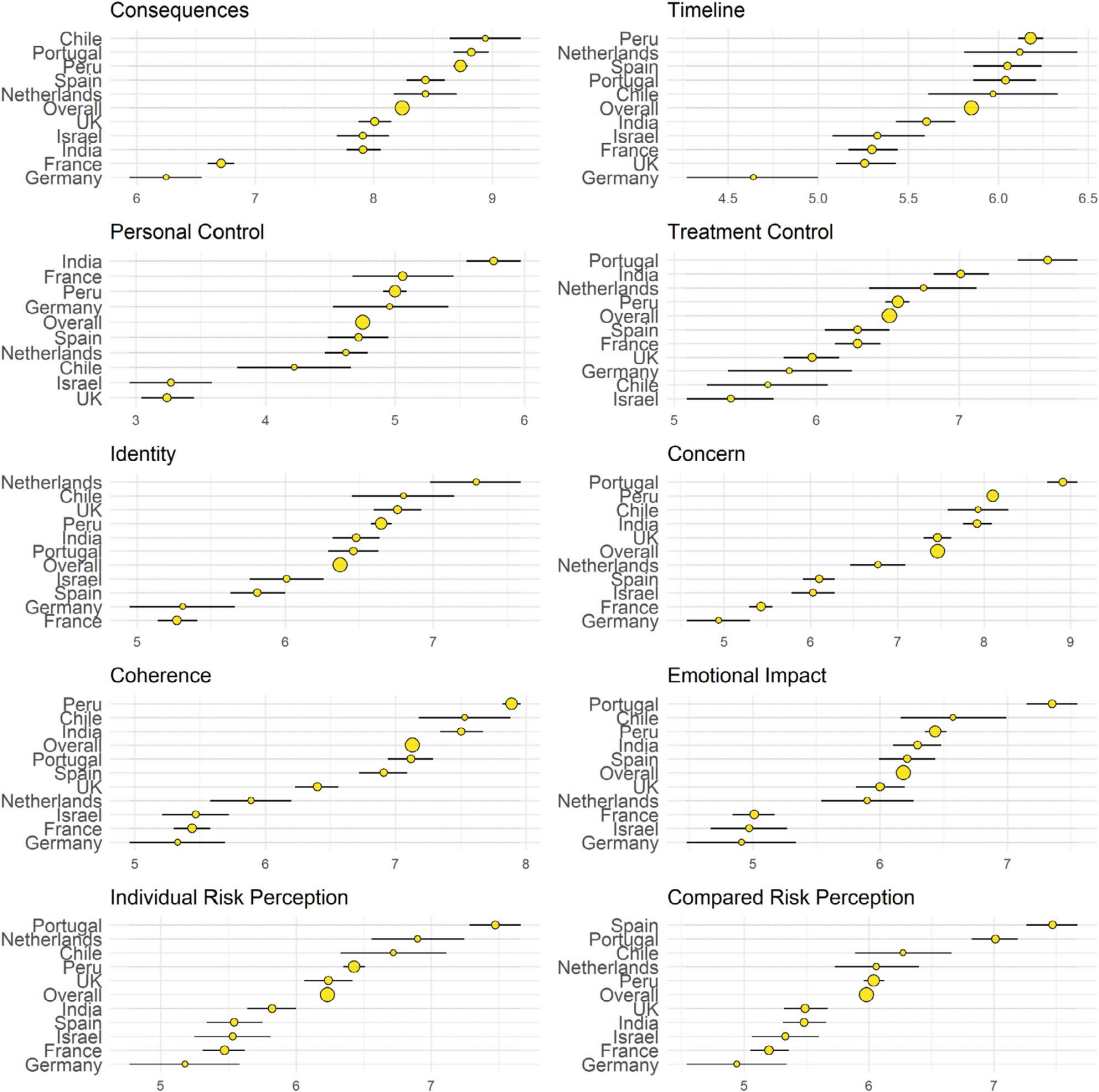
Means and 95% confidence intervals for each country in all B‐IPQ dimensions. Means and confidence intervals of each country in their scores of illness perceptions. Size of dots indicates sample size.

Regarding B‐IPQ item scores by country, high scores appeared in general (above 5 out of 10 in all items except one). Consequences were the highest item (around 8.25), followed by concern (around 7.5) and coherence (around 7). The lowest item was personal control (4.75), followed by timeline (around 5.8). In addition, participants perceived an intermediate level of personal control, while a medium‐to‐high level of treatment control. Thus, participants seem to perceive COVID‐19 as a serious disease, especially regarding how it affects infected persons, how concerned they are about it, and how well they feel to understand the illness. Nevertheless, participants perceived an intermediate personal control over COVID‐19, while they considered this disease to last an intermediate amount for infected people. In addition, participants perceived a medium‐to‐high risk of contagion overall and compared to those of the same age and sex. Regarding countries, France and Germany scored systematically lower than other countries in all items except for personal and treatment control. In those items, Israel, United Kingdom, and Chile had the lowest levels. Therefore, it seems that France and Germany displayed a generally less relevant or salient illness perception for COVID‐19 when compared to other countries. Finally, France and Germany were also the countries with the lowest perception of risk, expressing medium levels (around 5.5 in overall risk and around 5 in risk compared to same age and sex). Portugal displayed the highest levels of overall risk and the second highest in risk compared with those of the same age and sex. Spain displayed a surprising pattern, with medium levels of overall risk (around 5.5) and the highest level of risk compared to those of the same age and sex (around 7.5) (Figure 1).

**FIGURE 2:**
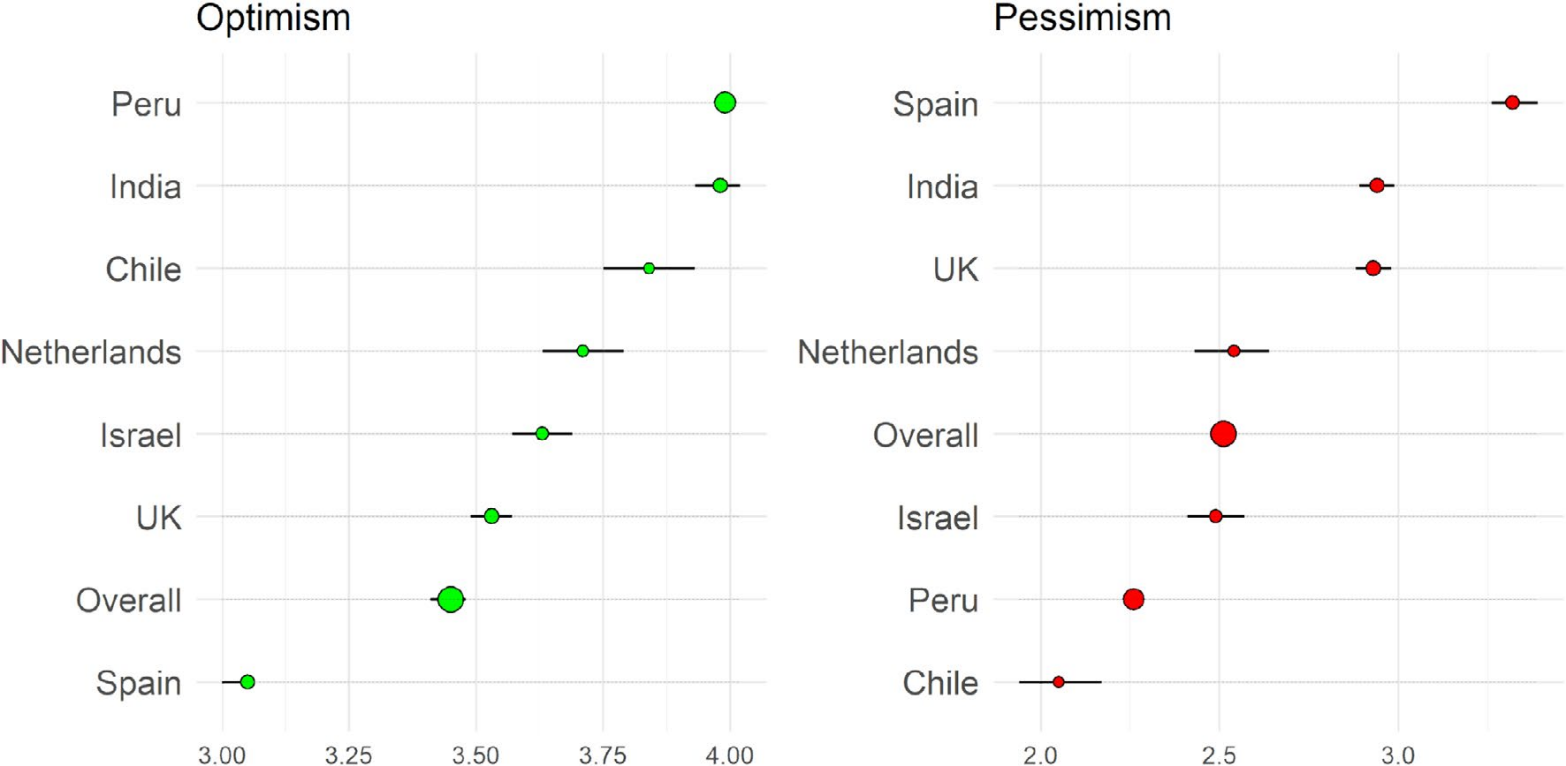
Means and 95% confidence intervals for each country in both LOT‐R dimensions. Means and confidence intervals of each country in their scores of optimism (green) and pessimism (red). Size of dots indicates sample size.

Regarding the LOT‐R scores by countries, average scores were obtained in overall samples, with slightly higher overall optimism scores (3.45) and slightly lower pessimism scores (2.51). India and Perú showed the highest optimism (almost 4) while Spain and the United Kingdom showed the lowest optimism (around 3 and 3.5, respectively). In addition, Spain and the United Kingdom showed the highest pessimism (around 3.3 and 3, respectively), while Perú and Chile showed the lowest pessimism (around 2.25 and 2, respectively) (Figure 2).

### 3.3 Associations Among Illness Perceptions, Absolute and Comparative Risk Perceptions, and Dispositional Optimism in Different Countries

Regarding MRAs (Table 3), B‐IPQ items displayed small explained variances (*R* ^2^ < 0.10) and optimism displayed significant positive associations with several B‐IPQ items. Optimism displayed its highest predictive coefficients with treatment control, personal control, and coherence. Thus, optimism seems to be associated with positive elements of illness prevention. However, optimism displayed small but significant positive associations with consequences, timeline, identity, concern, and compared risk perception. On the other hand, pessimism displayed its highest predictive coefficients for emotional impact, compared to risk perception and timeline. Thus, pessimism seems to be associated with negative aspects of illness perceptions, except for consequences. Regarding risk perception, optimism surprisingly displayed no significant negative coefficients. In contrast, it only showed a small but significant positive coefficient in relative risk perception. Pessimism showed significant positive coefficients in both absolute and relative risk perception (although small). The highest predictors were the presence of chronic disease (positive for absolute but negative in relative risk), having passed COVID‐19 (negative in both absolute and relative risk), and social isolation (also negative in both, although small).

**TABLE 3:** Standardized and unstandardized regression coefficients with 95% confidence intervals for each B‐IPQ item.

Variables	Consequence	Timeline	Personal control	Treatment control	Identity	Concern	Coherence	Emotional impact	Individual risk perception	Compared risk perception
Standardized coefficients (*β*) with 95% CI										
Optimism	0.06 [0.03; 0.09]	0.06 [0.03; 0.09]	0.18 [0.15; 0.21]	0.17 [0.15; 0.21]	0.06 [0.02; 0.09]	0.10 [0.07; 0.13]	0.16 [0.13; 0.19]			0.04 [0.01; 0.07]
Pessimism		0.09 [0.06; 0.12]				0.05 [0.01; 0.08]		0.18 [0.15; 0.21]	0.05 [0.02; 0.08]	0.10 [0.07; 0.13]
Age	0.07 [0.05; 0.10]	0.06 [0.03; 0.09]	−0.03 [−0.06; −0.01]		0.03 [0.01; 0.06]	0.04 [0.01; 0.08]				0.07 [0.04; 0.10]
Gender	0.10 [0.04; 0.16]		−0.06 [−0.11; −0.01]		0.09 [0.03; 0.14]	0.08 [0.02; 0.14]	−0.08 [−0.13; −0.02]	0.17 [0.11; 0.23]		
Chronic disease					0.30 [0.19; 0.41]	0.64 [0.44; 0.84]		0.27 [0.07; 0.48]	0.46 [0.26; 0.66]	−0.32 [−0.52; −0.12]
Number of days in lockdown			0.08 [0.05; 0.11]	0.06 [0.03; 0.09]			0.05 [0.02; 0.08]		−0.05 [−0.08; −0.02]	
Social isolation	−0.27 [−0.33; −0.20]	−0.23 [−0.30; −0.17]	−0.14 [−0.21; −0.08]			−0.26 [−0.32; −0.20]	−0.34 [−0.40; −0.27]	−0.24 [−0.30; −0.17]	−0.07 [−0.13; −0.01]	−0.16 [−0.23; −0.10]
COVID−19 infection: Self									−0.15 [−0.22; −0.07]	−0.20 [−0.28; −0.13]
COVID−19 infection: Familiar						−0.07 [−0.14; −0.02]	0.09 [0.03; 0.15]		−0.07 [−0.13; −0.01]	
COVID−19 infection: Friends										
Unstandardized coefficients (B) with 95% CI										
Optimism	0.19 [0.09; 0.28]	0.22 [0.10; 0.34]	0.87 [0.72; 1.02]	0.77 [0.63; 0.90]	0.21 [0.09; 0.32]	0.38 [0.26; 0.50]	0.60 [0.49; 0.72]			0.15 [0.02; 0.28]
Pessimism		0.26 [0.17; 0.35]				0.13 [0.04; 0.22]		0.59 [0.48; 0.69]	0.16 [0.06; 0.26]	0.31 [0.21; 0.41]
Age	0.01 [0.01; 0.01]	0.01 [0.01; 0.01]	−0.01 [−0.01; −0.01]		0.01 [0.01; 0.01]	0.01 [0.01; 0.01]				0.01 [0.01; 0.02]
Gender	0.18 [0.08; 0.28]		−0.17 [−0.33; −0.01]		0.19 [0.07; 0.31]	0.18 [0.06; 0.31]	−0.17 [−0.28; −0.02]	0.43 [0.29; 0.57]		
Chronic disease					0.66 [0.42; 0.90]	1.43 [0.99; 1.87]		0.70 [0.19; 1.22]	1.11 [0.62; 1.59]	−0.76 [−1.2; −0.29]
Number of days in lockdown			0.05 [0.03; 0.07]	0.04 [0.02; 0.05]			0.03 [0.01; 0.04]		−0.03 [−0.04; −0.01]	
Social isolation	−0.46 [−0.58; −0.35]	−0.52 [−0.66; −0.38]	−0.41 [−0.59; −0.23]			−0.58 [−0.72; −0.44]	−0.74 [−0.88; −0.60]	−0.61 [−0.77; −0.44]	−0.17 [−0.32; −0.01]	−0.39 [−0.55; 0.24]
COVID infection: Self									−0.35 [−0.53; −0.18]	−0.49 [−0.66; −0.31]
COVID infection: Familiar						−0.17 [−0.30; −0.03]	0.21 [0.08; 0.34]		−0.16 [−0.31; −0.02]	
COVID infection: Friends										
Adjusted *R* ^2^	0.03	0.02	0.05	0.03	0.01	0.07	0.07	0.03	0.01	0.05

*Note:* Only significant coefficients are displayed (*p* < 0.05). Compared Risk Perception = Risk of contagion compared with people of the same age and sex. Items of B‐IPQ are displayed on the OSF platform.

People with chronic diseases seem to perceive higher concern, individual risk perception, emotional impact, and identity for COVID‐19. Moreover, people practicing social isolation seem to perceive less understanding of COVID‐19, fewer consequences, concern, emotional impact, and duration of COVID‐19. In addition, they perceive less risk perception (both absolute and relative). Age showed several significant but small associations with B‐IPQ items since it seems to be a crucial risk factor for COVID‐19. Gender also showed several significant associations, but some with higher potential. Among them, the highest was the emotional impact (with women scoring higher than men). In addition, consequences and identity showed higher scores in women than in men.

## 4 Discussion

This study examines the role of optimism and pessimism on risk and illness perception during an unprecedented global crisis. Data were collected from 10 countries during the first wave of the pandemic, before vaccinations were available. During the first wave, when scientists had limited knowledge about the virus and vaccines were not available, people experienced high levels of concern and perceived negative consequences and coherence related to COVID‐19. Governments widely communicated that COVID‐19 was a new virus and its effects were only recently discovered (Zaim et al. 2020); we expected that it would be perceived as a strong threat. Our data indicate that participants perceived the first wave of COVID‐19 as a serious health‐threatening condition in 10 different countries. The findings also show that COVID‐19 was seemingly perceived across countries as a moderate (timeline) to severe (consequences, concern, coherence) disease with low personal control.

Regarding optimism, our results with a sample of 10 countries during the COVID‐19 pandemic seem to break with the idea that optimism displays a dysfunctional role in illness perception and risk assessment in the general population, and these patterns were consistent across all 10 countries. In general, studies show that critical situations such as the COVID‐19 pandemic affect optimism in the general population (Bottemanne et al. 2020; Hagger and Orbell 2003). In an uncertain and critical pandemic situation like the first wave of COVID‐19, optimism was hypothesized as a cognitive bias that could impact illness perceptions in a dysfunctional way (e.g., excessively low risk perception), among other psychosocial factors (Bottemanne et al. 2020; Dryhurst et al. 2020). This is an “unrealistic optimism” perspective. Countering these ideas, our findings across countries showed that optimism seems to be positively associated with treatment sense of control, personal sense of control, concern, and coherence (i.e., sense of understanding the disease). It is important to note that coherence measures the sense of understanding the disease (making the illness “coherent”), while concern measures a more emotional state of worry, personal sense of control measures the perception of how individuals can control the disease, and treatment control measures the sense of efficacy of medical treatment. These dimensions of illness perceptions represent beliefs about capacities to understand and manage/control the disease (Leventhal et al. 2016). As the findings were correlational, we propose three potential explanations: The first proposes optimistic individuals in a high uncertainty and low controllability situation as the first wave would show more intense beliefs of COVID‐19 as comprehensible and controllable by themselves (e.g., adhering to the only known preventive measures) and treatment (this is, the expectation of future vaccines at the time of the first wave of the pandemic). The second proposes that individuals with a higher sense of personal control, comprehension of the illness, and believing more intensely in the efficacy of future vaccines would show an increased optimistic outlook at the time of the first wave. The third one proposes a confound of individuals with higher positive functioning (e.g., higher eudaimonia well‐being, psychological virtues, or auto‐telic personality traits) being more predisposed to both more optimistic outlooks and functional management of information and preventive measures about COVID‐19, resulting in a higher sense of control. According to risk assessment, our results counter our hypotheses of negative associations between optimism and risk perception. Instead, optimism shows a potentially positive effect on risk perception, which counters a single previous study and not in general but in the clinical population (e.g., oncological patients regarding COVID‐19; Gabanelli et al. 2024). Recent studies conducted during the COVID‐19 outbreak found that optimism could have a protective effect on COVID‐19 stress and mental health (Arslan et al. 2021; Puig‐Pérez et al. 2021). Thus, we currently consider our first explanation to be more supported by evidence currently than the alternatives. This perspective can be described as “functional optimism.” Thus, optimism as a positive psychological resource may be an important variable to understand mental health during a world crisis such as the COVID‐19 pandemic (Arslan et al. 2021). If optimism contributes to reducing perceived stress in a high uncertainty and low controllability situation such as the first wave of the COVID‐19 pandemic, maybe it could also contribute to adapting risk and illness perceptions in a realistic way during the pandemic. Therefore, optimism may play a protective role in reducing distress produced not only by pandemic conditions (such as uncertainty or grief) through an increased sense of control and understanding the disease, but also by burdens derived from preventive measures (like social isolation or loneliness) (Puig‐Pérez et al. 2021).

In contrast, pessimism was associated mainly with a greater negative emotional impact caused by COVID‐19, while also expecting a greater timeline of COVID‐19. This finding suggests that a pessimistic outlook is associated with higher predictions of pandemic‐related distress and longer expected duration of the disease. As correlational findings, we draw three potential explanations. The first is that pessimism plays a cognitive bias toward negative information (e.g., their concern in a negative way), making the individuals increase their distress and suffering due to the pandemic. The second one is that the emotional impact and concern of the COVID‐19 pandemic (e.g., loss of loved ones, crippling medical consequences after being infected) increased a pessimistic outlook in people. And the third one relies on a confound of general psychological vulnerability. This is because individuals struggling with mental health issues would have damaged or destroyed psychosocial resources due to the pandemic (e.g., in‐person social interactions, distraction, or hobbies, contact with green spaces, physical exercise). And therefore, being more emotionally distressed and concerned. The validated versions of the LOT‐R exhibited discrepancies in number of items, as indicated in the instruments section. These variations may expose our results to minor differences in item content which limit our results. Future studies should test these differences with a psychometrically equivalent measure, as indicated in the limitation section.

In addition, we found moderate levels in illness perceptions regarding timeline. This finding suggests that people across countries perceived COVID‐19 as a non‐chronic disease. We interpret this as a reflection of transmitted information by media about COVID‐19 as an intense (and potentially deadly) flu. People could generalize the mechanisms and symptoms of the disease from their experience with ordinary flu. This finding also undermines the potential long‐term effects of COVID‐19 and could also explain the results on coherence (i.e., “How well do you feel you understand this illness?”), which displayed high scores in all countries in spite of being in the first wave yet. Also, we found high levels in consequences illness perceptions, suggesting people across countries perceived COVID‐19 as highly threatening. Note that this dimension measures the personal estimation of the negative impacts of the disease on their lives, functioning as a lack of clear and specific recommendations by scientists and institutions at the first wave of the pandemic. Regarding concern, we found that this dimension was rated high in all countries except Israel. Concern was also a strong predictor of distress (Dempster et al. 2015). The high emotional impact of uncertainties related to the new virus may generate an overreaction related to imprudent behavior and negative mental health consequences (Aven and Bouder 2020), contributing to adherence to social isolation and COVID‐19 measures (Chong et al. 2020). The low levels of personal control in all countries could be attributed to the uncertainty of the first wave of the pandemic. Many people felt they did not have enough information about the virus and, consequently, were unsure how they could cope with the situation (Dennis et al. 2021). For those people, the sense of personal control may decrease, as this dimension refers to the belief about how we can control the illness (Hagger and Orbell 2003). However, some variations across countries were found in literature, like high levels of personal control in the general Greek and Mexican population at the beginning of the pandemic (Skapinakis et al. 2020; Lugo‐González et al. 2020). Differences in these results may have occurred for methodological (using the full or brief forms of the IPQ‐R in their case, convenience online samples), cultural, or geopolitical reasons (e.g., different policies on preventive measures; Aven and Bouder 2020).

Interestingly, illnesses and risk perceptions differed from actual infection rates for each country during the first wave (e.g., https://ourworldindata.org/covid‐cases). For instance, Chile and Perú, while at medium or low‐risk perceptions in our data, had a high number of cases of infection and deaths per million (since May 30, 2020). Israel and India had a low number of cases per million, but the lowest number of deaths in this group of countries was in Germany and the Netherlands, which showed high risk perceptions in our data. India and Perú showed the highest optimism, while Spain and the United Kingdom showed the lowest optimism. Regarding Perú, it was the country most affected by COVID‐19 in the number of deaths in Latin American countries during the first wave, and its numbers are also high in terms of contagion and low testing. Therefore, subjective aspects of the pandemic experience do not necessarily correspond to objective infection rates and may be more related to how people experience social isolation and lockdown than rates of contagiousness and mortality because it is an individual experience. For example, some paradoxical effects of adherence to non‐pharmaceutical interventions have been noted; for instance, while body weight was a risk factor for a more severe COVID‐19 (Amin‐Chowdhury et al. 2021), it has also been shown that after the first lockdown there was an increase in body weight in the general population (Mason et al. 2021; Schippers 2020).

Some limitations need to be acknowledged. First, this online cross‐sectional survey limits the generalizability of the data and its causal effects. In addition, the sampling of data was by convenience sampling, while the countries were selected by convenience, which damages the generalizability of our findings. Future studies should aim for methods that enable causal claims, like longitudinal (e.g., ecologically momentary assessments) or experimental designs (e.g., randomized‐controlled trials). Instruments were all self‐reports, which exposes our findings to same‐method inflation bias. Future studies should aim to compare different instruments (e.g., biometric data). Some representativity concerns were present, like a majority of women. Future studies should aim for a representative sampling of countries and populations within them. The design was exploratory, which led to the risk of Type‐I errors. Future studies should aim for confirmatory, pre‐registered designs. Additionally, we did not measure the economic and social impacts of the pandemic, which could be one of the elements that concern the participants and impact their subjective perceptions of the illness. Future studies should aim to measure those aspects and compare them to illness perceptions and optimism.

## Author Contributions

Elisa Kern de Castro contributed to the conceptualization and design of the study, data collection, data interpretation, drafted the initial manuscript, and revised the final manuscript. Oscar Lecuona was involved in data collection, methodology, data analysis, data interpretation, drafted the initial manuscript, and revised the manuscript. Maria João Figueiras contributed to the conceptualization and design of the study, data collection, data interpretation and revised the manuscript. Cristina Quiñones contributed to the data collection, data analysis, data interpretation, and revised the manuscript. Kamlesh Singh contributed to the data collection, data analysis, data interpretation, and revised the manuscript. Shoshana Shiloh contributed to the data collection, data analysis, data interpretation, and revised the manuscript. Michaela Schippers contributed to the data collection, data analysis, data interpretation, and revised the manuscript. Ana Kinkead contributed to the data collection, data analysis, data interpretation, and revised the manuscript. Raquel Rodríguez‐Carvajal contributed to the conceptualization and design of the study, data collection, data interpretation, drafted the initial manuscript, and revised the final manuscript. Each author should review and approve the final manuscript and agree to be accountable for all aspects of the work.

## Ethics Statement

The research protocol was authorized by the Human Research Ethics Committee (HREC) of the Open University (HREC/3543).

## Conflicts of Interest

The authors declare no conflicts of interest.

## Data Availability

The data that support the findings of this study are openly available in OSF at https://osf.io/pdwj2/.
